# Overlap in processing advantages for minimal ingroups and the self

**DOI:** 10.1038/s41598-020-76001-9

**Published:** 2020-11-03

**Authors:** Florence E. Enock, Miles R. C. Hewstone, Patricia L. Lockwood, Jie Sui

**Affiliations:** 1 Department of Experimental Psychology, University of Oxford, Oxford, OX2 6GG UK; 2 Department of Psychology, University of York, York, YO10 5DD UK; 3 Wellcome Centre for Integrative Neuroimaging, University of Oxford, Oxford, UK; 4 Centre for Human Brain Health, School of Psychology, University of Birmingham, Birmingham, UK; 5 School of Psychology, University of Aberdeen, Aberdeen, AB24 3RT UK

**Keywords:** Psychology, Human behaviour

## Abstract

Cognitive biases shape our perception of the world and our interactions with other people. Information related to the self and our social ingroups is prioritised for cognitive processing and can therefore form some of these key biases. However, ingroup biases may be elicited not only for established social groups, but also for minimal groups assigned by novel or random social categorisation. Moreover, whether these ‘ingroup biases’ are related to self-processing is unknown. Across three experiments, we utilised a social associative matching paradigm to examine whether the cognitive mechanisms underpinning the effects of minimal groups overlapped with those that prioritise the self, and whether minimal group allocation causes early processing advantages. We found significant advantages in response time and sensitivity (*d*prime) for stimuli associated with newly-assigned ingroups. Further, self-biases and ingroup-biases were positively correlated across individuals (Experiments 1 and 3). However, when the task was such that ingroup and self associations competed, only the self-advantage was detected (Experiment 2). These results demonstrate that even random group allocation quickly captures attention and enhances processing. Positive correlations between the self- and ingroup-biases suggest a common cognitive mechanism across individuals. These findings have implications for understanding how social biases filter our perception of the world.

## Introduction

Information that is related to the self or a social ingroup to which one belongs is given high priority across a multitude of cognitive domains. Implicit self-associations tend to be positive^1,2^, people evaluate objects more favourably when they are given ownership of them^3–5^, participants are better at recalling adjective words that describe themselves^6^, and self-reference consistently enhances memory performance^7–9^. At the perceptual level, people show advantages in processing their own faces compared to those of other people^10^. Similarly, people generally prefer ingroups to outgroups^11^, with greater empathy shown towards ingroup members^12^, and implicit bias effects observed across a multitude of social contexts^13^. Group membership also influences lower-level perceptual judgements. Notably, there is a body of evidence demonstrating that social categorisation affects face processing. For example, biases are shown for own-race faces in recognition and memory^14^ and these effects extend to other social contexts too^15^. Thus, the cognitive prioritisation of self- and ingroup-related information, known as self-bias and ingroup-bias, is ubiquitous. However, less is known about whether the same cognitive mechanisms underpin prioritisation for the self and social ingroups.

While belonging to social groups is adaptively beneficial in providing protection, support and the sharing of important resources^16^ social categorisation is also a source of intergroup conflict and related issues such as prejudiced attitudes and discriminatory behaviours^17^. Understanding the cognitive basis of group membership and its relation to self identity therefore serves as an important foundation in guiding interventions to reduce, or even prevent, social conflict.

An associative matching paradigm developed by Sui and colleagues^18^ established a method to study social effects on cognitive prioritisation whilst controlling for complete equivalence across the target stimuli, a potential confound in previous studies of processing advantages that have often used face and name stimuli. In this paradigm, participants learned to associate geometric shapes (e.g., square, circle and triangle) with social labels (e.g., ‘you’, ‘friend’ and ‘stranger’). Then, participants completed a computerised task in which they were asked to judge whether shape-label pairs presented on the screen conformed to the original pairings or not by responding with keys for yes and no. The stimuli were presented quickly at 100 ms with a short response window of 1100 ms, allowing for automatic levels of processing. A large prioritisation effect was found in response time, accuracy and sensitivity scores for self shapes compared to those of friend and stranger, and for friend compared to stranger shapes. These effects have been robustly replicated^19–24^ and can operate even when associations are not instructed but are learnt over time^25^. Further, self-prioritisation exists even when familiarity is removed from all stimuli, such as when social labels were replaced with unfamiliar abstract symbols associated to the words (you, friend, stranger) before the experiment^26^. Initial studies have also demonstrated that ingroup associations can modulate processing for neutral stimuli in the same way^27–30^.

Whilst we tend to envision group membership as referring to static collectives imbued with historic meaning such as religions, nationalities and ethnicities, in fact the human drive for social classification is so great that ingroup favouritism is elicited even under ‘minimal group’ conditions, whereby individuals are assigned to novel groups randomly or on the ostensible basis of arbitrary information^31–35^. Interestingly, perceptual advantages for processing ingroup faces occur in minimal group contexts in the same way as for established groups^36^. This shows enhanced processing of own-group faces is not just rooted in greater exposure. There are competing theories as to how and why favouritism effects for minimal groups occur. According to Social Identity Theory^37^, individuals derive aspects of the self from the image of the ingroup, known as *self-stereotyping*. Ingroup favouritism occurs because when the group image is positive, self-esteem is enhanced by virtue of belonging. However, for self-stereotyping to occur, we would need adequate information about the group prototype, which would be unlikely in minimal group situations^38^. An alternative account for ingroup favouritism within the minimal group paradigm is known as *self-anchoring*^38,39^*,* which posits that rather than the self being assimilated to the group, it is the group that is assimilated to the self.

Studies that have examined the relations between self and ingroup biases have often used trait judgement paradigms which, for the most part, involve high-level evaluative decision-making processes^40^. To our knowledge, no studies have compared the relationship between self-bias and novel ingroup-bias for more automatic processes. Therefore it is unknown how these biases affect our processing of information in the world at an early stage. The primary aim of the present research was thus to test whether ingroup favouritism for minimal groups is demonstrated within low-level associative matching. To understand how group classification shapes earlier forms of processing has important implications for social biases at a multitude of levels. For example, biases in visual attention that are based in seemingly arbitrary criteria could provide the foundation for greater cognitive elaboration and in turn greater individuation of ingroup members. Correspondingly, lower attention to outgroups may lead to generalisation and deindividuation.

We examined whether neutral stimuli (lacking in any obvious social content) associated with novel ingroups are shown prioritisation compared to stimuli associated with novel outgroups. Secondly, we also examined how biases for novel ingroups relate to biases for the self. If self-anchoring is the means to favouritism for novel groups, then we may expect self-bias to be a reliable predictor of ingroup-bias. The paradigm utilised presently is beneficial in allowing for direct measurement and comparison of the magnitude of lower-level cognitive prioritisation for the self and novel ingroups under the same metric. This methodology provides a way of keeping complete equality between the familiarity of the target stimuli and the method of measuring responses to the stimuli.

Across three experiments we used a social associative matching paradigm to test for cognitive prioritisation of stimuli associated with novel ingroups, and to measure the relationship between these biases for the self and novel ingroups. In Experiment 1, all participants (within-subjects design) performed two separate matching tasks, one in which they learned to associate self and stranger labels with shapes (personal task), and the other in which they were allocated to novel teams and learned to associate ingroup and outgroup labels with different shapes (group task). In both tasks, participants had to respond to randomly presented shape-label pairs as correctly or incorrectly matched according to the learned associations. Experiment 2 aimed to examine the relationship between the self- and ingroup-prioritisation effects when the self, stranger, ingroup and outgroup associations were made within the same task. This allowed measurement of the relationship between the self- and ingroup-advantages when personal and group stimuli were salient in the same blocks of the same task, enabling us to test whether prioritisation for novel ingroups remains when the self is also salient. Experiment 3 measured the relationship between the self and ingroup advantages but this time when the personal and group social associations were made within different blocks of the same task. Performance advantages were always taken as the differences in response time and sensitivity (*d*prime) between own associated (self/ingroup) shapes and other associated (stranger/outgroup) shapes. Note that bias effects for the self and novel ingroups are also referred to interchangeably as prioritisation and advantage effects. We refer to self and stranger as personal associations and ingroup and outgroup as group associations throughout. We refer to self and ingroup as ‘own’ and stranger and outgroup as ‘other’. In Experiment 1 ‘task type’ refers to whether the task involved personal or group associations. In Experiments 2 and 3, this becomes ‘association type’ as the tasks are no longer separated into two. ‘Shape’ always refers to the specific social concept (e.g., ‘self’) that it represents according to the association made at the start of the experiment. Mismatch trials are defined by shape such that mismatched trials for the self condition, for example, refer to a self shape presented with a stranger label. We analysed our data by shape condition (as opposed to label) because our primary interest is in how social meaning can be quickly tagged to novel stimuli (i.e., the shapes), rather than in biases for already learned social content (i.e., social labels).

We hypothesised that (1) A robust performance advantage for the novel ingroup would be demonstrated on the associative matching task in the same way as for the self and (2) If there is an overlap in the way we process information about the self and minimal ingroups, there should be a positive relationship between biases for the self and the novel ingroup.

## Experiment 1—associative matching of self and novel ingroups

### Methods

#### Ethics

All studies (1–3) were approved by the Ministry of Defence Research Ethics Committee (MODREC) (approval number: 495/MODREC/13) and informed consent was obtained prior to the experiment according to approved ethical procedures. All studies were performed in accordance with the relevant guidelines and regulations.

#### Participants

Participants were recruited via a university online recruitment system. In a related previous research study^28^ (Experiment 1), a sample of forty-two gave power of 0.99 to detect a significant effect of shape on response time and sensitivity (alpha 0.05) with partial eta^2^ of ~ 0.60 in an similar repeated-measures design (power calculation conducted using MorePower 6.0.4). A similar sample size was planned in order for direct comparisons to be drawn. A total of fifty individuals took part (the additional eight participants were recruited to allow for data exclusion and drop out). Three were excluded on the basis of having chance accuracy for one or both tasks (M < 55%), leaving a total of forty-seven (24 female, range 19–34 years, mean age = 23.6, SD = 3.66). All were right-handed and had normal or corrected-to-normal vision.

#### Stimuli and tasks

**Figure 1:**
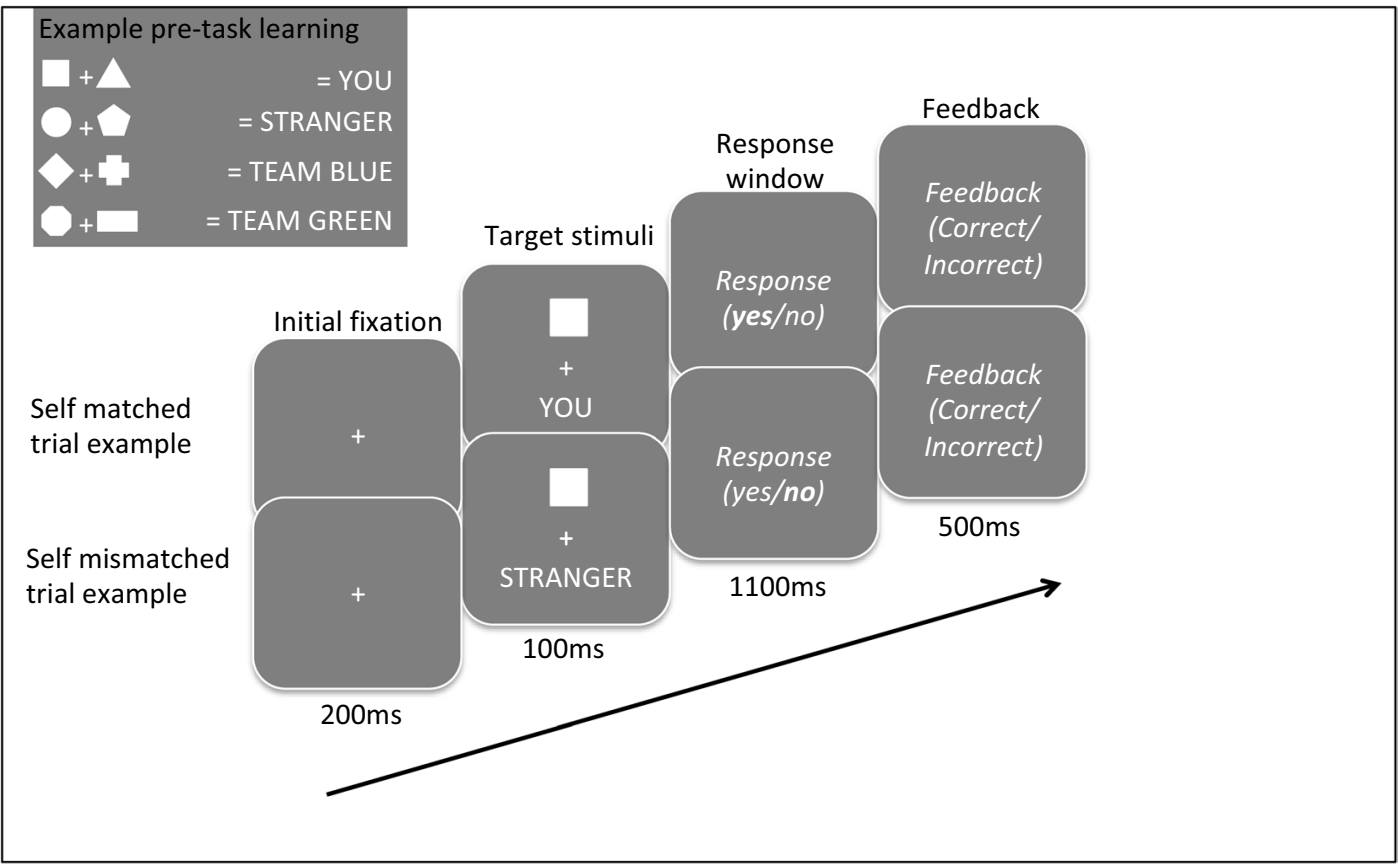
Self and ingroup associative matching paradigm. A schematic representation of the matching task that was utilised across all three experiments. The shape-label pairings were always counterbalanced across participants. After a short learning phase of the initial shape-label associations, participants responded to randomly presented shape-label pairs shown on-screen as matched or mismatched by pressing assigned response keys. In Experiment 1, the personal (you and stranger) associations were made in a separate task to the group (ingroup and outgroup) associations and order of tasks were counterbalanced. In Experiment 2, all of the associations (personal and group) were learned and presented in the same blocks of the same tasks. In Experiment 3, the associations were learned as part of one task, but were presented and responded to in alternating blocks. Two shapes were associated with each label in Experiments 1 and 3 (as shown in the figure), but only one shape was associated with each label in Experiment 2.

Two associative matching tasks adapted from Sui et al.^18^ were performed by each participant in a within-subjects design (Fig. 1). Each task took approximately twelve minutes. In the personal task, participants learned to associate two geometric shapes with ‘self’ and two with ‘stranger’ labels and then had to judge if randomly presented shape-label pairs were correctly or incorrectly matched. The group task took the same format, but participants learned to associate two geometric shapes with novel ingroup and outgroup labels (Team Green and Team Blue, counterbalanced). Although word length and category were not made equivalent, control experiments in previous work verified self bias effects were not rooted in word length, concreteness, usage frequency or familiarity^18,29,41^. Thus, we were confident we could choose the simplest words to identify own and other categories without impacting results.

For both tasks, the shape-label pairings were initially made by an on-screen instruction (e.g., ‘you are the square and the triangle and the stranger is the pentagon and the circle’). The associations were displayed at the same time and stayed on the screen until participants pressed the spacebar to continue to the experiment. By this design, the shape-label mappings are not learned during the course of the experimental task, but are rather explicitly instructed at the start. After these initial associations were made, 12 practice trials were completed and then a 2-s central fixation cross indicated the start of the main computer task. In each trial, a randomly-generated shape-label pair was shown on screen for 100 ms. Then, there was a 1100 ms blank response window in which participants indicated whether the pair had been correctly or incorrectly matched by pressing *m* or *n*, counterbalanced for *yes* or *no*. Feedback was given for 500 ms after the response for each trial. In total, 384 experimental trials were spread over 6 blocks (64 trials per block). Accuracy was presented at the end of each block followed by an 8-s break. There were four conditions: two match conditions, (matched/mismatched); and two shape conditions (self and stranger), with 96 trials per condition. The task descriptions are based on methods described in Enock et al.^28^ for Experiment 1.

The associations across both tasks between the eight shapes (circle, triangle, square, pentagon, octagon, cross, diamond, rectangle) and the four written labels (self, stranger; Team Blue, Team Green) were counterbalanced across participants. Order of task completion was also counterbalanced such that half the participants completed the personal task first and half completed the group task first. In both tasks, the geometric shapes (each 118 × 118 pixels, 4.16 × 4.16 cm, corresponding to a visual angle of around 4.8° × 4.8° as viewed 50 cm from the screen) were presented randomly above a white fixation cross (0.8° × 0.8°) against a grey background at the centre of the screen, with a matched or mismatched label presented simultaneously below the central fixation point. The experiment was run on a PC using E-Prime software (version 2.0) and displayed on a 17-inch monitor.

#### Procedure

Upon arriving to the experimental session, informed consent was obtained in accordance with approved ethical procedures and participants were briefed with regards to what the computer tasks involved. Then, participants were verbally allocated to either Team Green or Team Blue by being told:

*‘For this study, each participant is randomly assigned to one of two teams: Team Green or Team Blue. You are on Team (Green). Please remember your team affiliation throughout the study’.*

Those allocated to Team Blue received the corresponding instruction. Team allocation was made such that odd-numbered participant numbers were allocated to Team Green, while even-numbered participant numbers were allocated to Team Blue. After group allocation, some brief demographic information was taken and participants began the first of the two computer tasks. The first screen showed the shape-label pairings and once participants felt confident they had learned them, they pressed the spacebar to continue to the first block. When participants had completed the first task, they were given a short break while the researcher prepared the computer for the next part, and when they were ready, they began the second task. At the end, participants were debriefed and remunerated for their time at a rate of £10 per hour.

#### Statistical analyses

For all experiments, data were analysed in SPSS 24 (Armonk, New York: IBM Corp). For all response time analyses, only correct responses were included, and those higher or lower than 2.5 standard deviations from the mean response time for each participant under each condition were excluded as a standard way of removing outliers^42^. Less than 5% of each dataset was excluded and each analysis was performed on the remaining trials. Data were also analysed using signal detection theory (dprime)^43^ which is a useful measure that allows for the distinction between effects that can be attributed to changes in perceptual sensitivity (i.e., the ability to discriminate between targets) and effects that are related to response biases (i.e., whether participants have a preferred type of response throughout the task). The estimation of parameters included requires the classification of each response type as either a correct identification of a target (a ‘hit’ response), a correct rejection of a non-target (a ‘correct rejection’), a misidentification of a non-target as a target (a ‘false alarm’) and misjudging a target as a non-target (a ‘miss’). In the current experimental design, a response was considered as a hit when a matching shape-label pair appeared and participants responded ‘yes’; a correct rejection when the shape and label did not match and participants responded ‘no’; false alarm when a shape and label did not match and participants responded ‘yes’; and a miss when a shape and label matched but a ‘no’ response was given. The sensitivity index (*d*prime) represents the ability to distinguish matching and shape-based mismatching stimuli, and the response criterion represents a bias towards judging mismatching stimuli as matching, or the opposite. Matched and mismatched trials for each shape were combined to give a measure of *d*prime using the following formula, where H denotes correct hits and F denotes false alarms^43^:

$${d}^{\prime}=z\left(\mathrm{H}\right)-z(\mathrm{F})$$

and the response criterion (RC) for each participant was also calculated as follows^44^

$$RC= -0.5*[z\left(\mathrm{H}\right)+z(\mathrm{F})]$$

For each experiment, we examined biases for the self and ingroup using repeated measures ANOVAs on response time and sensitivity (*d*prime) scores. There were two task types (personal: self and stranger associations; and group: ingroup and outgroup associations), two shape conditions (own: self and ingroup; and other: stranger and outgroup); and two match conditions (matched/mismatched). Note: ± always denotes standard error of the mean.

## Results (Experiment 1)

### Own-associated biases in response time data

There were three within-subject variables: task type (personal: self/stranger or group: ingroup/outgroup associations); shape category (own: self/ingroup, or other: stranger/outgroup) and match condition (matched/mismatched). A 2 (task: personal/group) × 2 (shape: own/other) × 2 (matched/mismatched) repeated-measures analysis of variance (ANOVA) was used to test for the effect of shape on response time scores.

There was no significant effect of task type, *F*(1, 46) = 0.90, *p* = 0.347, η_p_^2^ = 0.019, showing that response time was similar across the personal and group tasks. There was a significant effect of shape, *F*(1, 46) = 29.62, *p* < 0.001, η_p_^2^ = 0.392, with faster response times for own (*M* = 745 ± 8.59) than other (*M* = 766 ± 8.64) shapes. There was also a significant effect of match condition, *F*(1, 46) = 242.73, *p* < 0.001, η_p_^2^ = 0.841, with faster response times for matched (*M* = 725 ± 8.54) than for mismatched (*M* = 786 ± 8.68) trials.

There was no significant interaction between task and shape, *F*(1, 46) = 1.01, *p* = 0.319, η_p_^2^ = 0.022, nor between task and match, *F*(1, 46) = 1.54, *p* = 0.222, η_p_^2^ = 0.032. There was, however, a significant interaction between shape and match condition *F*(1, 46) = 58.09, *p* < 0.001, η_p_^2^ = 0.558, and there was also a significant three-way interaction between task, shape and match, *F*(1, 46) = 8.24, *p* = 0.006, η_p_^2^ = 0.152. To decompose these interactions, separate 2 (task type) × 2 (shape) ANOVAs were performed for matched and mismatched trials. For matched trials, there was no significant main effect of task type, *F*(1, 46) = 1.55, *p* = 0.220, η_p_^2^ = 0.033, but there was a significant main effect of shape, *F*(1, 46) = 62.51, *p* < 0.001, η_p_^2^ = 0.576, and a significant interaction between the two, *F*(1, 46) = 6.53, *p* = 0.014, η_p_^2^ = 0.124. Self shapes (*M* = 699 ± 10.05) were responded to significantly faster than stranger shapes (*M* = 763 ± 8.44), *t*(46) = 7.99, *p* < 0.001, *d* = 1.16, and ingroup shapes (*M* = 698 ± 11.78) were responded to significantly faster than outgroup shapes (*M* = 741 ± 11.23), *t*(46) = 5.57, *p* < 0.001, *d* = 0.81.

**Figure 2:**
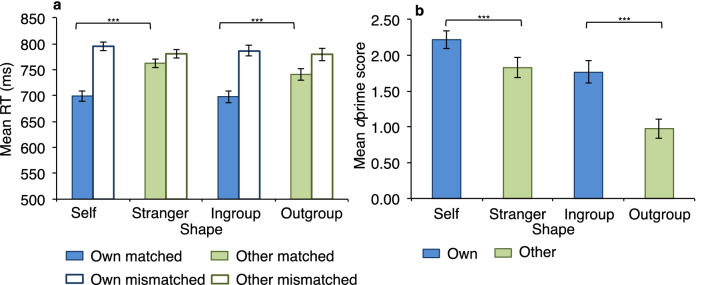
Self and ingroup information is prioritised and processed with greater sensitivity compared to strangers and outgroups. Mean response time (RT) scores as a function of shape (self vs. stranger, ingroup vs. outgroup), match (matched vs. mismatched) and task (self vs. group) **(a)** along with mean sensitivity (*d*prime) scores as a function of shape and task **(b)** for Experiment 1. Error bars represent standard errors. *** denotes *p* < .001; * denotes *p* < .01; n.s. denotes non-significant.

For mismatched trials, there was no significant main effect of task type, *F*(1, 46) = 0.33, *p* = 0.571, η_p_^2^ = 0.007, but there was a significant main effect of shape condition, *F*(1, 46) = 5.32, *p* = 0.026, η_p_^2^ = 0.104, with other shapes responded to significantly faster than own shapes. There was no significant interaction between task type and shape condition, *F*(1, 46) = 0.33, *p* = 0.298, η_p_^2^ = 0.024. In summary, the effect of shape on response time was similar across both tasks. Figure 2a shows response times for matched and mismatched trials for each shape association.

### Own-associated biases in dprime (perceptual sensitivity)

A 2 (shape: own/other) × 2 (task: personal/group) repeated measures ANOVA revealed a significant main effect of shape, *F*(1, 46) = 46.63, *p* < 0.001, η_p_^2^ = 0.503 and of task type, *F*(1, 46) = 24.44, *p* < 0.001, η_p_^2^ = 0.347. Sensitivity was greater for own (*M* = 1.99 ± 0.12) than for other (*M* = 1.40 ± 0.11) shapes, and for the personal (*M* = 2.02 ± 0.11) than the group (*M* = 1.37 ± 0.13) task.

There was a significant interaction between shape and task, *F*(1, 46) = 7.70, *p* = 0.008, η_p_^2^ = 0.143. Self shapes were responded to with greater sensitivity than stranger shapes, *t*(46) = 3.68, *p* < 0.001, *d* = 0.54, and ingroup shapes were responded to with greater sensitivity than outgroup shapes, *t*(46) = 6.72, *p* < 0.001, *d* = 0.98, with the latter effect almost twice the magnitude. Additionally, response criterion scores were significantly lower for self than stranger shapes and for ingroup than outgroup shapes (both *p* < 0.01). Similar to response time data, the *d*prime scores demonstrated performance advantages for self compared to stranger, and for ingroup compared to outgroup. Figure 2b shows sensitivity scores for each shape association.

## Discussion (Experiment 1)

The first experiment aimed to establish whether there was an ingroup-advantage in associative matching within the minimal group paradigm. For matched trials, self shapes were responded to significantly faster than stranger shapes and ingroup shapes were responded to significantly faster than outgroup shapes. For mismatched trials, stranger shapes were responded to significantly faster than self shapes, but there was no difference in response time between ingroup and outgroup shapes. Slower responses for self than stranger on mismatched trials is a common finding within this paradigm and the self-bias effect is usually only observed within matching trials^18^. *D*prime scores were enhanced for self over stranger and for ingroup over outgroup shapes. Further, there was a lower response criterion for self and ingroup than stranger and outgroup shapes, showing participants tended to more readily indicate that the stimuli were own- than other-associated. In line with our main hypothesis, these results demonstrate that an ingroup-bias in attentional prioritisation is manifested even when group affiliations are new, arbitrary and randomly assigned, and in the absence of other group members.

These results complement the body of literature demonstrating the effects of minimal group allocation on both higher-level decision-making ^31,32,35^, and also on more implicit processes^45–47^. The present results show novel group allocation affects the earlier processing of previously neutral, non-face stimuli. This experiment benefitted from a unique approach to measuring minimal group-biases by using random ingroup allocation combined with an associative matching paradigm, useful in allowing for comparison across social associations whilst keeping the measurement and visual features of the stimuli exactly the same.

Experiment 2 included self, stranger, ingroup and outgroup associations within the same task blocks so we could test whether self- and ingroup-biases remain constant when both types of stimuli are salient at the same time. As this paradigm included combinations of shape-label pairings for self and ingroup together, this also allowed us to examine the overlap between self and novel ingroup representation. For example, higher error rates and slower response times for self shapes paired with ingroup labels than self shapes paired with outgroup labels would indicate a cognitive overlap between self and ingroup stimuli.

## Experiment 2—competition between self and ingroup

### Methods

#### Ethics

As before, ethics was approved by MODREC (approval number: 495/MODREC/13) and all participants gave informed consent. Relevant guidelines and regulations were followed.

#### Participants

Participants were recruited via the university online system. Sample size was again planned according to a related previous research study^28^ (Experiment 2) in which an N of thirty-one gave power of 0.97 to detect a significant effect of shape on response time and sensitivity (alpha 0.05) with partial eta^2^ of 0.346 for response time (0.484 for *d*prime) in a similar repeated-measures design. Using these effect sizes as estimates, a sample size calculation showed eighteen participants would be sufficient to detect the effect of shape on response time and sensitivity with power of 0.8 and alpha 0.05. Therefore, although this sample size was smaller than that of Experiment 1, a larger sample size was not necessary to establish the effects of interest. The power and sample size calculations were conducted using MorePower 6.0.4. A total of 22 individuals (14 female, age range 19–28 years, mean age = 23.3, *SD* = 2.71) took part, all right-handed and with normal or corrected-to-normal vision.

#### Stimuli, task and procedure

The task was very similar to those in Experiment 1, but this time the personal and group associations were made within the same blocks of the same matching task rather than separately in two tasks. Other than that, exactly the same experimental parameters were used as for Experiment 1. In an initial on-screen instruction, participants learned to pair Self, Stranger, Team Green and Team Blue labels with different geometric shapes (circle, square, triangle, pentagon), fully counterbalanced across participants. All shape-label associations were displayed together and remained on the screen until participants pressed spacebar to continue. In each trial, participants again judged whether shape-label pairings presented on the screen were correctly or incorrectly matched. The task consisted of six blocks of 96 trials per block (excluding 12 practice trials at the beginning). There were eight conditions within the task: four shape conditions (self, stranger, ingroup, outgroup) and two match conditions (matched/mismatched), with 72 trials per condition. The procedure followed exactly that of Experiment 1, except participants completed one rather than two tasks.

#### Statistical analyses

In addition to the analyses we conducted in Experiment 1, we compared specific combinations of personal and group mismatched pairs in response time and accuracy in order to test whether participants found it more difficult to reject self and ingroup paired stimuli than self and outgroup or ingroup and stranger. A greater difficulty in rejecting self and ingroup as correct would indicate an overlap in the cognitive representation of newly learned self and ingroup associations. Paired samples t-tests were used to examine differences in response time and accuracy scores for *shape* + label mismatches of *self* + ingroup and *self* + outgroup, along with *ingroup* + self and *ingroup* + stranger.

### Results (Experiment 2)

#### Own-associated biases in response time data

A 2 (association type: personal/group) × 2 (shape: own/other) × 2 (matched/mismatched) repeated measures ANOVA found a significant effect of association type, with responses faster for the personal (*M* = 682 ± 10.89) than the group (*M* = 705 ± 11.01) shapes, *F*(1, 21) = 25.06, *p* < 0.001, η_p_^2^ = 0.544. There was a marginal effect of shape, *F*(1, 21) = 4.23, *p* = 0.052, η_p_^2^ = 0.167, with faster response times for own (*M* = 687 ± 10.86) than for other (*M* = 698 ± 10.91) shapes. There was a significant effect of match condition, *F*(1, 21) = 79.28, *p* < 0.001, η_p_^2^ = 0.791, with responses faster for matched (*M* = 663 ± 9.60) than for mismatched (*M* = 725 ± 12.71) pairs.

There was no significant interaction between association type and shape, *F*(1, 21) = 2.70, *p* = 0.116, η_p_^2^ = 0.114. However, there was a significant interaction between association type and match condition, *F*(1, 21) = 25.01, *p* < 0.001, η_p_^2^ = 0.544, and between shape and match condition, *F*(1, 21) = 18.56, *p* < 0.001, η_p_^2^ = 0.469. There was also a significant three-way interaction between association type, shape and match condition, *F*(1, 21) = 7.38, *p* = 0.013, η_p_^2^ = 0.260.

To decompose this, separate 2 (association type) × 2 (shape) ANOVAs were performed for matched and mismatched trials. For matched trials, there was a significant main effect of association type, *F*(1, 21) = 38.39, *p* < 0.001, η_p_^2^ = 0.646, with faster responses for the personal (*M* = 637 ± 9.96) than the group (*M* = 688 ± 10.90) task. There was also a significant main effect of shape, *F*(1, 21) = 17.18, *p* < 0.001, η_p_^2^ = 0.450, with faster responses for own (*M* = 646 ± 10.41) compared to other (*M* = 679 ± 10.38) shapes. Importantly, a significant interaction between association type and shape, *F*(1, 21) = 6.57, *p* = 0.018, η_p_^2^ = 0.238, showed that for matched trials, responses were significantly faster for self than for stranger shapes (*p* < 0.001) but not for ingroup than outgroup shapes (*p* = 0.356).

**Figure 3:**
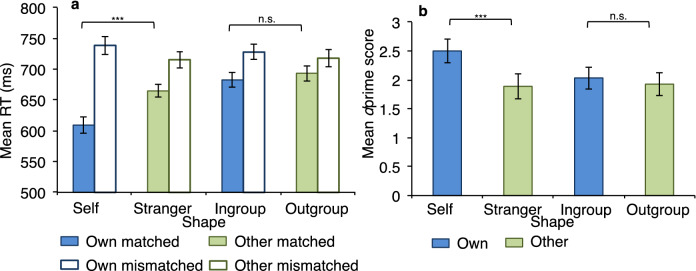
Self, but not ingroup, information is prioritised and processed with greater sensitivity. Mean response time (RT) scores as a function of shape (self vs. stranger, ingroup vs. outgroup), match (matched vs. mismatched) and task (self vs. group) **(a)** along with mean sensitivity (*d*prime) scores as a function of shape and task **(b)** for Experiment 2. Error bars represent standard errors. *** denotes *p* < .001; * denotes *p* < .01; n.s. denotes non-significant.

For mismatched trials, there was no significant effect of association type, *F*(1, 21) = 0.49, *p* = 0.494, η_p_^2^ = 0.023, with similar response times for personal (*M* = 727 ± 13.67) and group (*M* = 722 ± 12.42) trials. There was a significant main effect of shape type, *F*(1, 21) = 7.89, *p* = 0.011, η_p_^2^ = 0.273, with faster responses for other (*M* = 717 ± 12.92) than own (*M* = 733 ± 13.17) shapes. There was no significant interaction between association type and shape condition *F*(1, 21) = 1.63, *p* = 0.216, η_p_^2^ = 0.072. In summary, responses were significantly faster for self than stranger shapes (but not for ingroup than outgroup) on matched trials, while for mismatched trials, responses were overall faster for other than own associations. Figure 3a shows response times for matched and mismatched trials under each shape condition.

#### Own-associated biases in dprime (perceptual sensitivity)

A 2 (association type: personal/group) × 2 (shape: own/other) repeated measures ANOVA found no significant effect of association type, *F*(1, 21) = 3.22, *p* = 0.087, η_p_^2^ = 0.133, with comparable scores for personal (*M* = 2.19 ± 0.19) and group (*M* = 1.98 ± 0.19) associations. There was a significant effect of shape, *F*(1, 21) = 8.20, *p* = 0.009, η_p_^2^ = 0.281, with greater sensitivity for own (*M* = 2.27 ± 0.18) than for other (*M* = 1.91 ± 0.20) shapes. A significant interaction, *F*(1, 21) = 5.63, *p* = 0.027, η_p_^2^ = 0.211, indicated the effect of shape was dependent on the association type, with enhanced perceptual sensitivity for self shapes relative to stranger shapes *(p* = 0.005), but not for ingroup relative to outgroup shapes, (*p* = 0.417).

Similar to response time data, sensitivity was enhanced for self relative to stranger, but not for ingroup relative to outgroup stimuli. Additionally, the response criterion was significantly lower for self shapes than for stranger shapes (*p* = 0.002), but there was no difference in response criterion between ingroup and outgroup shapes (*p* = 0.124). Figure 3b shows sensitivity scores for each shape condition.

### Analysis of mismatched pairs

#### Response time data

Although there was no significant advantage for the novel ingroup compared to the novel outgroup, performance for specific mismatched shape-label combinations tested an overlap between the representation of the self and novel ingroup. If the novel ingroup is anchored to the self, then we would expect slower response times when responding ‘no’ to a self shape mismatched with an ingroup label than a self shape mismatched with an outgroup label, as the latter would be more obviously incongruent. Paired-samples t-tests were conducted to determine if there were differences in response time scores between self shapes paired with ingroup labels and self shapes paired with outgroup labels. There were 24 trials for each mismatched condition.

There were no significant differences in response times for self shapes paired with ingroup labels than for self shapes paired with outgroup labels, *t*(21) = 1.22, *p* = 0.235, *d* = 0.26. However, ingroup shapes paired with self labels were responded to significantly faster than ingroup shapes paired with stranger labels, *t*(21) = 2.19, *p* = 0.041, *d* = 0.46. That participants were faster to correctly reject ingroup shapes paired with self labels is opposite to the predicted effect, and merely suggests generally faster responses for self labels than stranger labels.

#### Accuracy data

Accuracy scores were significantly higher for self shapes paired with outgroup labels than self shapes paired with ingroup labels, *t*(21) = 2.18, *p* = 0.041, *d* = 0.47. Accuracy scores were not significantly higher for ingroup shapes paired with stranger labels compared to ingroup shapes paired with self labels *t*(21) = 1.87, *p* = 0.075, *d* = 0.40. That participants found it easier to reject self + *outgroup* than self + *ingroup* may indicate an overlap in the way that newly learned self and novel ingroup stimuli are represented. Table 1 shows the mean response times and proportions of correct responses (accuracy scores) for each mismatched pair of interest.

**Table 1:** Mean response times for correct responses and accuracy rates (proportions of correct responses) for specific mismatched pairs (Experiment 2). Standard deviations are in parentheses.

Shape + label	RT (ms)	Accuracy
Self + ingroup	745 (79.0)	0.72 (0.24)
Self + outgroup	731 (74.5)	0.79 (0.16)
Ingroup + self	707 (691.1)	0.79 (0.21)
Ingroup + stranger	729 (57.6)	0.86 (0.13)

### Discussion (Experiment 2)

Experiment 2 tested whether self- and novel ingroup-advantages in associative matching are present when shape-label pairs were shown within the same blocks of the same task. This design allowed for comparison of self and ingroup biases when both kinds of social stimuli were present at the same time. A strong self-bias effect was found, with significantly faster responses for self than for stranger shapes on matched trials (but not on mismatched trials) and significantly enhanced *d*prime scores. However, there was no response time or *d*prime advantage for ingroup compared to outgroup shapes. Similarly, there was a response criterion bias for self shapes compared to ingroup shapes, with participants more likely to indicate stimuli as self- than stranger-associated, but with no such bias for ingroup compared to outgroup shapes. These results contrast to those from Experiment 1, which demonstrated a robust ingroup-advantage for new and arbitrarily assigned groups. Similarly, the results contrast to prior work in which a significant ingroup-advantage (with a similar sample size) in the context of well-established teams was found even when the self and ingroup pairings were presented within the same task blocks^28^. The results are, however, consistent with a recent study showing minimal ingroup prioritisation was lower in magnitude than self-prioritisation when the two kinds of social stimuli were salient at the same time, and barely present when other ingroup members were not known at all^27^, as was the case in the present study. These findings suggest that novel ingroup-biases do not follow exactly the same patterns as those for established groups, particularly when competing with the saliency of the self.

The results imply that when self and novel ingroup stimuli are presented at the same time, the self takes priority, with the ingroup-bias effect disappearing. Thus, in a more cognitively demanding task, self significance plays a more important role in performance than minimal group affiliation. Interestingly, this is not the case for established groups, in which both kinds of social biases remained constant even in the presence of one another^28^. Turner’s principle of functional antagonism, which is an important part of self-categorisation theory and closely linked to the social identity perspective^48^, holds that whilst many social identities exist within each individual, as the salience of one increases, the saliency of the other will correspondingly decrease. Thus if the self is salient in comparison to the minimal group, then attention may be shifted entirely to the self. With regards to the notion of self-anchoring, it may be that when explicit self stimuli are present, the projection of the self onto novel ingroup associations is less important and the experimental stimuli become categorised more as ‘self’ or ‘non-self’.

Although there was no ingroup-advantage, responses to specific combinations of shape-label mismatches provided some evidence for greater difficulty in distinguishing self from ingroup stimuli. Error rates were lower for self shapes paired with outgroup labels than with ingroup labels which indicates a closer association between self and the minimal ingroup, than self and the minimal outgroup. This may suggest a self-anchoring mechanism in which the novel ingroup is incorporated to the self^39^. Results from Experiment 2 were not consistent with those of Experiment 1 and did not support our main hypothesis that minimal ingroups would be prioritised over outgroups in the associative matching task. Experiment 3 aimed to test this discrepancy in a similar sample size to Experiment 2 by separating self and ingroup associations into alternating blocks to reduce competition between self and ingroup whilst still controlling for practice and order effects.

## Experiment 3—blocked associative matching design

### Methods

#### Ethics

As before, ethics was approved by MODREC (approval number: 495/MODREC/13), all participants gave informed consent and relevant guidelines and regulations were followed.

#### Participants

As for Experiments 1 and 2, participants were recruited via the online system. Based on the sample size calculation described for Experiment 2, and for a comparable sample size, a total of 22 individuals took part (12 female, age range 19–35 years, mean age = 25.2, *SD* = 4.5). All of the participants were right-handed and had normal or corrected-to-normal vision.

#### Stimuli, task and procedure

The task was very similar to that for Experiment 2, but this time the personal and group associations were made within alternating blocks of the same task and participants learned to associate two shapes per label rather than one. Other than that, exactly the same experimental parameters were used. In an initial on-screen instruction, participants learned to pair two geometric shapes each with Self, Stranger, Team Green and Team Blue labels, again counterbalanced. All shape-label associations were displayed together and remained on the screen until participants pressed spacebar to continue. In each trial, participants had to judge whether shape-label pairings subsequently presented were correctly or incorrectly matched. There were six blocks of 80 trials per block (excluding 12 practice trials at the beginning) and personal and group blocks were alternated such that there were 3 of each. Half of the participants were presented with a personal block first and half with a group block first. Participants were given a break in between each block during which they were reminded on-screen of the shape-label associations. There were eight conditions: four shapes (self, stranger, ingroup, outgroup) each either matched or mismatched, with 60 trials per condition. The procedure was the same as for Experiment 2.

### Results (Experiment 3)

#### Own-associated biases in response time data

A 2 × 2 × 2 repeated measures ANOVA with association type (personal/group), shape condition (own/other) and match condition (matched/mismatched) as the three within-subject variables was used to test for the effect of shape on response time scores. There was no effect of task type on response time, *F*(1, 21) = 0.04, *p* = 0.847, η_p_^2^ = 0.002, showing that response times were generally similar for personal and group associations. There was a significant effect of shape on response time, *F*(1, 21) = 24.17, *p* < 0.001, η_p_^2^ = 0.535, with faster responses for own (self and ingroup) (*M* = 722 ± 14.9) than other (stranger and outgroup) (*M* = 756 ± 14.2) shapes. There was also a significant effect of match condition, *F*(1, 21) = 64.09, *p* < 0.001, η_p_^2^ = 0.753, with responses to matched pairs (*M* = 714 ± 14.0) significantly faster than responses to mismatched pairs (*M* = 764 ± 14.9).

**Figure 4:**
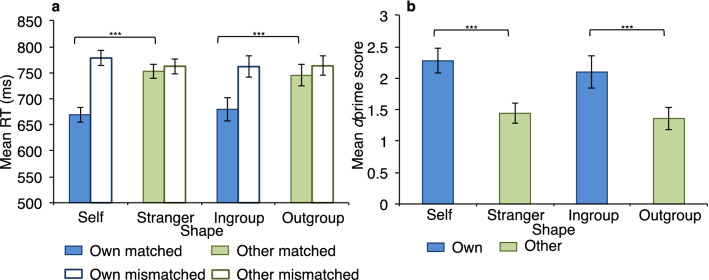
Self and ingroup stimuli are prioritised over stranger and outgroup and responded to with greater sensitivity. Mean response time (RT) scores as a function of shape, match and task **(a)** and mean sensitivity (*d*prime) scores as a function of shape and task **(b)** for Experiment 3. Error bars represent standard errors. *** denotes *p* < .001; n.s. denotes non-significant.

There was no significant interaction between task and shape, *F*(1, 21) = 0.004, *p* = 0.950, η_p_^2^ = 0.000, but there was a significant interaction between task and match condition, *F*(1, 21) = 4.35, *p* = 0.049, η_p_^2^ = 0.171, and between shape and match condition, *F*(1, 21) = 68.78, *p* < 0.001, η_p_^2^ = 0.766, with own-associated shapes responded to faster than other-associated shapes on matched (*p* < 0.001) but not mismatched (*p* = 0.059) trials (Fig. 4a). There was no significant three-way interaction between task, shape and match, *F*(1, 21) = 0.39, *p* = 0.540, η_p_^2^ = 0.018.

#### Own-associated biases in dprime (perceptual sensitivity)

A 2 (association type: personal/group) × 2 (shape: own/other) repeated measures ANOVA found a significant effect of shape on sensitivity scores, *F*(1, 21) = 43.95, *p* < 0.001, η_p_^2^ = 0.677, with sensitivity significantly enhanced for own (self and ingroup) (*M* = 2.19 ± 0.20) over other (stranger and outgroup) (*M* = 1.40 ± 0.15) shapes (Fig. 4b). There was no significant effect of association type on sensitivity, *F*(1, 21) = 0.92, *p* = 0.349, η_p_^2^ = 0.042, and no significant interaction between association type and shape, *F*(1, 21) = 0.15, *p* = 0.710, η_p_^2^ = 0.007. Overall, sensitivity was significantly enhanced for self relative to stranger shapes and for ingroup relative to outgroup shapes. As before, a significantly lower response criterion was adopted for own compared to other shapes (*p* < 0.01).

### Discussion (Experiment 3)

Experiment 3 examined self and minimal ingroup advantage effects when the social associations were separated into alternating blocks within the same task. The ingroup advantage in response time and sensitivity that was observed in Experiment 1, but not in Experiment 2, was re-established and there were similar effect sizes for the self and ingroup response time and sensitivity advantages. These results were consistent with those from Experiment 1 and were also in line with our main hypothesis of a minimal group advantage effect in the associative matching paradigm. These results again complement the wider literature showing that minimal groups are prioritised across a range of cognitive processes. The self and ingroup bias effects in response time were observed only in the matched trials which, as noted in the discussion for Experiment 1, is consistent with previous work using this paradigm.

Although the self and ingroup associations were present within the same task as for Experiment 2, the separation into alternating blocks appeared to sufficiently reduce competition for attention between the self and novel group. This suggests individuals are able to quickly switch attentional priority between self and novel ingroup relevance when they are required to only focus on one at a time. Overall, this experiment provides further evidence for an ingroup-advantage for novel social categories.

### Is there overlap between self and ingroup biases?

#### Analysis plan

Results from Experiments 1 and 3 showed that response times (for matched pairs) were enhanced for own (self and ingroup) shapes compared to other (stranger and outgroup) shapes. Correlation analyses were used to measure the relationship between the self- and ingroup-biases in order to elucidate whether shared mechanisms drive these prioritisation effects. As *d*prime scores combine matched and mismatched trials and the main bias effects occurred only for matched trials, only response time data was used for the subsequent analyses.

The data from Experiments 1 and 3 were combined because the pattern of results was identical. Difference in response times between self and stranger and between ingroup and outgroup were taken as a measure of the magnitude of the self- and ingroup-biases. Only matched trials were included in calculating the bias scores for response time because the self- and ingroup-advantages in response time were only observed within these. Scores for own shapes were deducted from scores for other shapes (as lower scores indicate better performance) and the relationships between the self- and ingroup-advantages were then measured using Pearson bivariate correlations.

Two data-points were identified as showing extremely low self- and ingroup-bias scores (below 2.5SD from the group mean) in response time. These outliers were excluded for the following analysis^49,50^. The total *N* for the subsequent analyses was 67.

### Results

**Figure 5:**
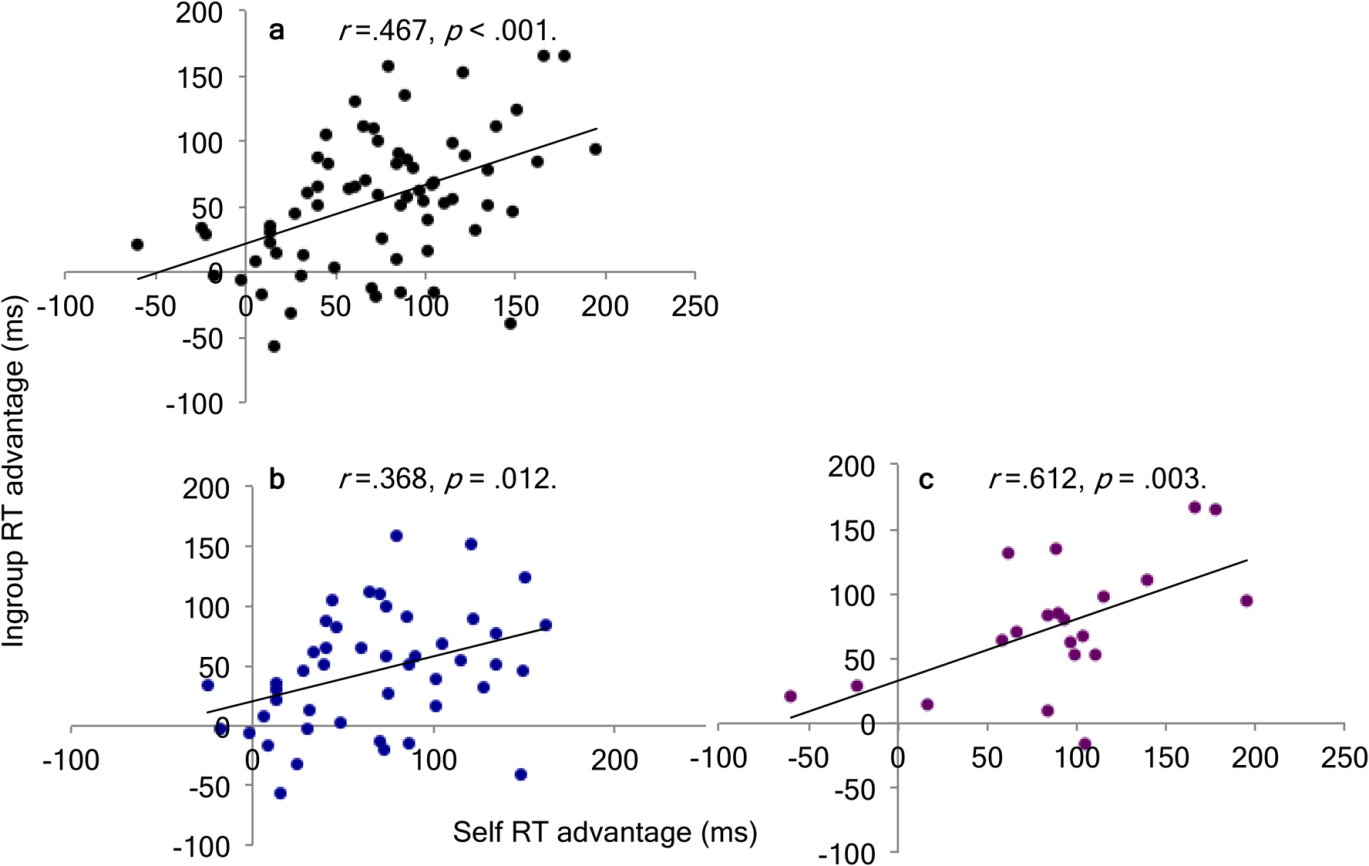
Overlap in processing advantages for the self and social ingroups. Pearson bivariate correlations showed a positive relationship between self and ingroup advantages in RT across Experiments 1 and 3 combined **(a)**; Experiment 1 **(b)** and Experiment 3 **(c)**.

There was a significant positive relationship between the self- and ingroup-advantages in response time, *r*(65) = 0.467 [0.275, 0.636], *p* < 0.001. When split into the two separate experiments, this effect held for both Experiment 1, *r*(44) = 0.368 [0.097, 0.599], *p* = 0.012, and also Experiment 3, *r*(19) = 0.612 [0.323, 0.819], *p* = 0.003 (Fig. 5).

## General discussion

Across three experiments, we examined the effects of minimal group allocation on low-level biases in associative matching. A robust advantage in response time and sensitivity for stimuli associated with the self and the ingroup was found when self and ingroup associations were responded to in separate tasks (Experiment 1) and in separate blocks of the same task (Experiment 3). However, when self and ingroup associations were presented within the same task blocks, the prioritisation effects were only observed for stimuli associated with the self (Experiment 2). Participants also tended to adopt a lower response criterion for own shapes compared to other shapes, showing they were generally more likely to indicate a shape was own- than other-associated in Experiments 1 and 3, although this bias only held for personal associations in Experiment 2. There was no ingroup-advantage effect when self and ingroup stimuli were present in the same task blocks, but higher error rates for self shapes paired with ingroup than with outgroup labels indicated the possibility of a shared cognitive representation of newly-learned self and (novel) ingroup stimuli and show a closer association between self and novel ingroup than self and novel outgroup. Further to this, there were positive correlations between the self and ingroup response time biases in both Experiments 1 and 3, suggesting the possibility of a self-anchoring mechanism driving the prioritisation effects for new social ingroups.

As noted throughout these experiments, the prioritisation effects found for novel ingroup stimuli within the matching task complement several studies that find ingroup favouritism effects in the minimal group paradigm across a range of outcome measures^51^. Notably, the results extend previous research showing processing advantages for novel ingroup faces^36^. Our results demonstrate that minimal group-bias extends even when no obvious social content (e.g., faces) is present. It may be that this fundamental bias in categorising social information may serve as the foundation for higher-level ingroup bias effects (e.g., a perceptual model of intergroup relations^52^). For example, greater cognitive resources allocated to ingroup stimuli may then cause better differentiation of ingroup and outgroup members in facial processing. The present findings significantly broaden these empirical findings and suggest even minimal group allocation provides a motivating factor in directing attention and enhancing cognitive performance.

Experiments 1 and 3 demonstrated a minimal group effect but this was eliminated when self stimuli were present within the same task blocks (Experiment 2). An ingroup-bias was demonstrated previously in the same design as Experiment 2 using an established group manipulation of college rowers^28^. This discrepancy indicates a possible difference in the mechanisms behind novel and established ingroup-prioritisation. It is likely that established social groups contain inherently more meaning and personal significance than novel ones which enables them to remain a priority even when self stimuli are also present. Novel groups, however, may not hold enough weight to retain attention when task demands increase and the self competes for attention. This is supported in a recent study^27^, which found significantly reduced ingroup-prioritisation compared to self-prioritisation when groups were randomly and arbitrarily assigned with no information given about the other group members. This point highlights an important methodological consideration: if novel group-biases do not always follow the same patterns as established group-biases, then studies utilising minimal group paradigms hold the potential of showing a different pattern of responses. It would be useful for further research to delve into the specific mechanisms behind the processing of established and novel ingroup stimuli.

Both Experiments 1 and 3 provided evidence for a positive relationship between the self- and novel ingroup-advantage in response time, indicating the possibility of a shared process driving the advantage effects. Although conclusions that can be drawn are limited in that data is correlational only, this is consistent with experimental evidence and theory finding ingroups are represented as part of the self^48,53–55^. This dovetails with work on self-anchoring^38,39^, which suggests ingroup favouritism arises, at least in some cases and particularly in the case of minimal groups, by the projection of the self onto the group, contrasting to the traditional social identity approach, which explains ingroup-bias in terms of self-stereotyping^37^. It is important to note that prior work found no relationship between biases for the self and high reward or positive valence^56,57^ when measured by an associative matching paradigm in a similar way. Thus, it is not likely that results are explained simply by individual differences in effectiveness of prioritisation systems more generally as there do not appear to be relationships between self-bias and other goal-relevant categories. The relationship observed presently, then, implies a conceptual connection between self- and novel ingroup-prioritisation.

Original research for self-anchoring utilised a trait judgement paradigm in which it was found that participants ascribed characteristics to novel ingroups based on information about the self^39^. The present study also implies that ingroup-bias arises from the projection of self significance. This may serve as a cognitive heuristic that allows a gap to be filled from the known (the self) to the unknown (the novel ingroup)^38^.

The three experiments benefitted from replicating previous findings using a minimal group paradigm in which self- and ingroup-prioritisation measures were exactly equal. However, there are some limitations to the design. It must be noted that in response time data for Experiments 1 and 3, it is difficult to completely disentangle effects of shape and label for mismatching pairs. This is because, for example, a stranger mismatched shape is by definition presented with a self label and a self mismatched shape is presented with a stranger label. Thus, it could be argued that faster responses for stranger than self mismatched shapes could be interpreted simply as faster responses for self than stranger labels. However, our dprime data strengthen the importance of interpreting results by shape (not label), as here match and mismatch trials by shape are combined to give a measure of overall sensitivity towards the shape. Additionally, in Experiment 2, mismatching shapes may have been presented with any one of the possible three mismatching labels. Prior research too provides grounds for focussing on the importance of shape category over label. For example, one study found biases for ingroup shapes when they were presented on sequential screens to label cues, ensuring responses were to shapes and not labels^29^. Another more recent study found self bias effects in the complete absence of familiar labels. Here, labels were replaced with abstract symbols before the experimental task started^26^, showing self bias effects did not depend on familiar social labels. Future work could employ these approaches to further separate the role of social labels in minimal group designs.

## Conclusions

Here we provide novel evidence for minimal group effects on the processing of neutral stimuli. Further, positive relations between self- and novel ingroup-biases provide support for self-anchoring: the projection of the self onto novel social groups to fill a cognitive gap between the known and the unknown. These findings could have important implications for understanding how social importance biases our perception of the world. Even arbitrary group allocation can quickly alter subsequent attentional allocation of stimuli associated with those new groups, and this could have consequences for many real-world social contexts in which categorisation occurs.

## Data Availability

Data will be made available on request.
